# Stem Cell Statute in Jordan: Leading the Way

**DOI:** 10.3389/fgene.2020.00657

**Published:** 2020-07-17

**Authors:** Amal Al-Tabba’, Rana Dajani, Maysa Al-Hussaini

**Affiliations:** ^1^Office of Human Research Protection Program, King Hussein Cancer Center, Amman, Jordan; ^2^Department of Biology and Biotechnology, Hashemite University, Zarqa, Jordan; ^3^Jepson School of Leadership, University of Richmond, Richmond, VA, United States; ^4^Department of Pathology and Laboratory Medicine, King Hussein Cancer Center, Amman, Jordan

**Keywords:** stem cell, statute, Islam, Jordan, research

## Abstract

The use of stem cells in research has caused much controversy and ethical dilemma. The primary source of stem cells is human embryos, a source which has been confronted with objections based on ethical, moral, and religious positions. Jordan has passed the first of its-kind Statute in the region, aiming at regulating the use of stem cells both for therapeutic and research purposes. The Statute adopted a regulatory approach between the restrictive and intermediate. The Statute, however, pays more attention to stem cell banking in many of its articles. Many critical aspects in regulating stem cell research activities are overlooked. This is including but not limited to the process of informed consent, protecting privacy, maintaining confidentiality, the need for a national entity responsible for regulating embryonic stem cell (ESC) research, and requirements of monitoring activity. The authors recommend further review of the current Statute in light of the deficiencies discussed so as to develop a more comprehensive and coherent Statute.

## Introduction

“The power of regenerative medicine,” “The greatest scientific health breakthrough in 100 years” and so many other attractive promises counting on stem cells as a tempting therapy for a continuously expanding list of medical disorders including neurodegenerative disease, diabetes mellitus, and heart disease, to name only a few. However, the use of stem cells has raised lots of controversies and ethical debates. The primary source of stem cells is the human embryo; a source that has been confronted by objections based on moral, ethical, and religious grounds (Fadel, 2012).

In countries with a Muslim majority, bioethics is highly dependent on and typically inseparable from religious jurisprudence. Legislation and bioethics principles in many Islamic countries are based on four sources of Islamic law collectively known as Shari’ah: the Holy Quran, Sunnah, Ijma’ (Consensus), and Qiyas (Analogy) (Al-Bar and Chamsi-Pasha, 2015a).

During the Islamic World League meeting held in Makkah al-Mukarramah, Saudi Arabia, in 2003, the Islamic Fiqh Council has agreed that it is permitted to obtain, grow, and use stem cells for therapeutic or permissible scientific research, if the source of the specimen is legitimate (Muslim World League, 2020). Most Muslim scholars agree that human life beings somewhere between 40 or 120 days after conception (Walters, 2004; Al-Aqeel, 2009; Dajani, 2014). While there is unanimous opinion that human life after ensoulment must not be transgressed against, difference of opinion exists when exactly ensoulment takes place, and with regard to the sanctity of the embryo prior to this phase.

Reproductive and therapeutic cloning shares many of the same techniques; nonetheless, they differ in their purpose. Reproductive cloning aims to create an identical copy of a human, whereas therapeutic cloning aims to produce embryonic stem cells (ESCs) to restore injured or diseased tissue or to be used in research (National Human Genome Research Institute, 2017). Ultimately, in March 2005, the 191 U.N. member states agreed to prohibit the use of human reproductive cloning (UNGAOR, 2005). Similarly, the Decree by the International Islamic Fiqh Academy of the Organization of Islamic Conferences (OIC-IFA) in Jeddah, during its 10th session in 1997, forbade all methods of human cloning that lead to human reproduction (Al-Aqeel, 2005).

## Stem Cells Laws

Stem cell laws and policies regulate research, sources, and uses of stem cells in humans including reproductive and therapeutic cloning, ESC research, and cord blood banks. They vary significantly by country and have raised many concerns and controversies. A survey of national ESC and human cloning policies in 50 countries worldwide reported unanimous banning of reproductive cloning of human beings (45 by national laws and five by national guidelines). Many also prohibit human cloning for therapeutic or research purposes (32 by national laws and five by national guidelines) (Isasi and Knoppers, 2006a). Yet, it has been noted that 21 countries have no clear policy on ESC research. Most of the countries that have adopted public policy to permit the use of ESCs or gametes in research are governed by strict conditions (16 countries by national laws and seven by national guidelines). The remaining countries clearly prohibit the use of embryos in research (Isasi and Knoppers, 2006a).

In the past decade, the Arab World has witnessed a dynamic change in stem cell research, whereby several countries are proactively supporting stem cell research by seeking funding initiatives, building research facilities, and creating research programs (El-Awady, 2008; Yahia, 2013; Abumaree et al., 2014). A very recent example is a stem cell center in the United Arab Emirates (UAE), where researchers are developing a treatment for COVID-19 using stem cells to regenerate damaged lung cells (Al Arabiya English, 2020). Moreover, the number of cord blood banks has grown remarkably (Table 1). This could be related to factors such as increased disease incidence and decreased fertility rates (Matsumoto et al., 2015).

**TABLE 1:** Cord Blood Banking Options in the Arab World (Matsumoto et al., 2015).

**Bank**	**Type**	**Storage Location**	**Collection Office Location(s)**
BabyCord	Private	United States (Boston)	Jordan (Amman)
Biovault Family	Private	United Kingdom (Plymouth)	Lebanon, UAE
CellSafe	Private	Egypt (Cairo)	Egypt
Cells4Life	Private	United Kingdom (Burgess Hill, Essex)	Bahrain, Egypt, Jordan, Kuwait, Lebanon, Qatar, Saudi Arabia, UAE
Center for Stem Cell Research and Regenerative Medicine	Public	Egypt (Assiut)	Egypt
Cryo-Save	Private	Priv UAE (Dubai), Belgium (Niel)	Egypt, Kuwait, Oman, Saudi Arabia, UAE
DCRC	Hybrid	UAE (Dubai)	UAE
Future Health Biobank	Public	United Kingdom (Nottingham), Switzerland (Châtel-St-Denis)	Bahrain, Egypt, Jordan, Kuwait, Lebanon, Morocco, Qatar, Saudi Arabia, Syria, UAE
KAIMRC	Public	Saudi Arabia (Riyadh)	Saudi Arabia
KFSH-RC	Public	Saudi Arabia (Riyadh)	Saudi Arabia
KHCC	Public	Jordan (Amman)	Jordan
Precious Cells	Private	United Kingdom (Middlesex)	Jordan, Lebanon, UAE
Smart Cells	Private	United Kingdom (West Drayton)	Egypt, Jordan, Kuwait, Lebanon, Syria, UAE
Sultan Qaboos Univ. Hospital	Oman (Muscat)	Public	Oman
Virgin Health Bank	Private, Hybrid	Qatar (Doha)	Qatar

Despite this, only Saudi Arabia, Egypt, Tunisia, and Algeria have a few non-comprehensive stipulations regulating the use of stem cells in research, according to a report in 2011 (UNESCO Cairo Office, 2011). In 2012, the Supreme Council of Health in Qatar has developed a set of permissible policies for stem cell research, which has not been legally enforced to this date (Wadvalla, 2012). In 2015, Saudi Arabia have issued a second edition of its Law of Ethics of Research on the Living Creature (originally issued in 2010) to include ethics of stem cells research (National Committee of BioEthics [NCBE], 2016; Editor and Edwards, 2017).

**FIGURE 1:**
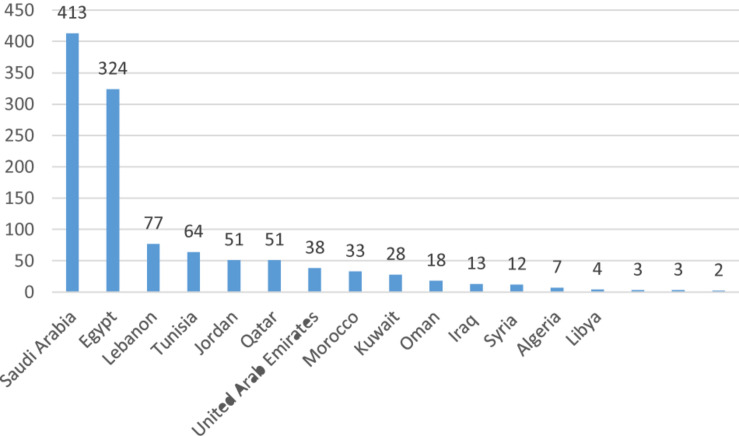
Stem cells published articles in the Arab World from 2005 to 2014 (Arab Scientific Community Organization, 2014).

**FIGURE 2:**
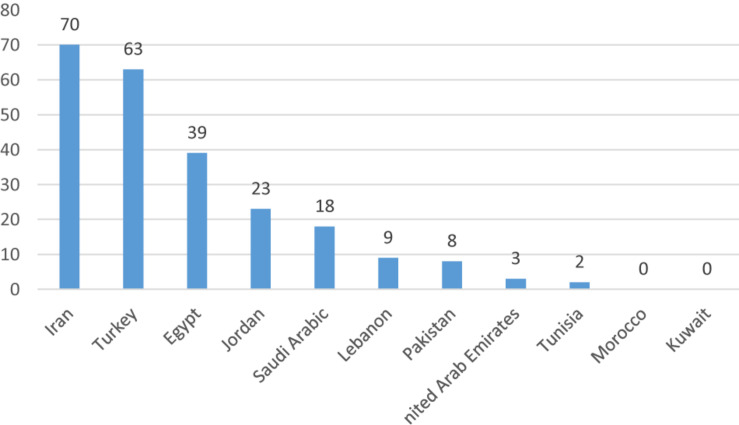
Stem cells clinical trials in the Greater Middle East, 2019 (unpublished data).

It is noted that most stem cell research activities in the Arab World are being conducted in the absence of any regulatory policies and/or public discussions (Flynn and Matthews, 2010). A special piece published in Nature Middle East in 2013 revealed that despite challenges to reach consensus on ethical polices of stem cell research in many Arab Countries, researchers are still pursuing stem cell research and attempt to become leaders in stem cell therapy, especially in diseases most prevalent in the region (Yahia, 2013). A study in 2010 identified 51 published articles on stem cells from seven different Arab countries over a 10-year period (1998–2008). These countries included: Saudi Arabia (*n* = 19), Egypt (*n* = 12), UAE (*n* = 6), Lebanon (*n* = 4), Tunisia (*n* = 4), Kuwait (*n* = 3), Morocco (*n* = 2), and Jordan (*n* = 1) (Flynn and Matthews, 2010). Another study in 2014 identified 1141 published articles on stem cells from the Arab World in the period from 2005 to 2014 with Saudi Arabia (*n* = 413) and Egypt (*n* = 324) being the biggest contributors (Figure 1) (Arab Scientific Community Organization, 2014). The number of clinical trials using stem cells retrieved from www.clinicaltrials.gov in June 2019 is shown in Figure 2 (unpublished data).

Scientists in these countries usually depend on religious rulings to guide and outline permissible practices. These rulings, however, lack the necessary structure to nurture international collaborations with countries having adopted formal laws (Gottweis et al., 2009; Flynn and Matthews, 2010). The presence of national stem cell research policies is directly related to a country’s stem cell research output. Hence, national regulation plays a pivotal role not only in regulating stem cell research activities and protecting the welfare of the public, but also in determining the productivity of stem cell researchers (Levine, 2008).

## The Jordanian Stem Cell Statute (JSCS)

Before the inception of the statute, and in line with other Islamic countries, the norm was for religious scholars to meet and make decisions concerning matters of jurisprudence that relate to all issues of life. In 2008, one of the co-authors (RD) took a novel approach by establishing a committee composed of multiple stakeholders, including physicians, scientists, Arabic language experts, and religious scholars, to contemplate and explore issues related to stem cell therapy and research along with associated social and ethical issues. The committee created a list of the issues related to stem cell therapy and research, and each issue was then examined to determine if consensus exists. If consensus existed, the committee either agreed or reassessed the verdict. In the absence of consensus, the issue was studied further (Dajani, 2017). The findings of this committee were presented at the “Belief in Dialogue” conference organized by the British Council in 2011 (Dajani, 2011).

In parallel, a body of scientists and physicians led by the Director of the Cell Therapy Center at the University of Jordan actively lobbied with government officials to create a Statute to guide stem cell therapy and research. The urgency was a result of the establishment of the first stem cell regenerative therapy center in Jordan. The Science and Technology Ethics Committee and the Ministry of Education were also consulted. The Statute was finally approved in 2014 by the General Iftaa’ Department of Jordan (the council of Muslim scholars) (Iftaa’ Department, 2019a) before its enactment (Dajani, 2014). In 2014, by the authority of the King of Jordan, Statute number 10 on Stem Cells was issued under Law number 47 on Public Health (Article 6) (The Hashemite Kingdom of Jordan, 2014).

Accordingly, the aim of this paper is to provide an analytical overview of the content of the JSCS and to explore its comprehensiveness and consistency. Based on a literature review of published articles, reports, and guidelines on stem cells research ethics, regulations, and policies, the authors identified the most commonly addressed ethical elements for stem cell research regulations. Consequently, five ethical elements were derived from the literature including legal status, regulatory approach, substantive requirements, procedural and ethical safeguards, and requirements for research governance. Each of the five ethical elements and its subsections were discussed briefly. Article(s) in the JSCS addressing each ethical element were highlighted. Additionally, gaps in any of the ethical elements, not covered by the JSCS, were specified and recommendations were made to narrow such gaps.

### Legal Status

Legislation governing stem cell research is often hard to adopt; however, setting rational and consistent ethical frameworks, with adequate safety and quality safeguards should be possible (Isasi and Knoppers, 2006a, b). The JSCS permits the use of stem cells for research including the restricted use of ESCs if specific criteria are met, based mainly on the fatwa of the General Iftaa’ Department of Jordan (The Hashemite Kingdom of Jordan, 2014).

### Regulatory Approach

In 2006, Isasi and Knoppers created a three-level classification of the regulatory approaches to ESC and cloning research adopted in 50 countries worldwide. These are restrictive, intermediate, and liberal (Isasi and Knoppers, 2006a).

Using this classification, we advocate that the JSCS falls between the restrictive and intermediate approaches. The restrictive approach prohibits many techniques through strict regulations or absolute bans, whereas the intermediate approach allows a wide range of techniques that are controlled and closely monitored. For example, under the intermediate approach, stem cell research on supernumerary embryos from *in vitro* fertilization (IVF) treatment is permitted (Isasi and Knoppers, 2006a). Aiming to provide efficient and safe mechanisms for conducting research, the JSCS (Article 3) allows the use of supernumerary embryos before a defined cut-off date (i.e., 5 days after fertilization), which is similar to a restrictive approach (The Hashemite Kingdom of Jordan, 2014).

### Substantive Requirements

The majority of international policies have emphasized the importance of the research goals in promoting scientific advancement and development of human interests as well as improving human well-being/health and relieving human suffering (Isasi and Knoppers, 2006a).

The need for justification for embryo use and/or destruction in research should always be provided. Substantive requirements include: (a) the importance of the research aims and rationale and (b) the method used to achieve such aims (Isasi and Knoppers, 2006b). Nonetheless, finding a balance between legitimate purposes and research aims, stem cell sources, and the conditions under which they have been produced or obtained, may be difficult to achieve, resulting in contradicting policies from a legal and ethical perspective.

Article (7) of the JSCS outlines substantive requirements for the permissible use of stem cells: (a) it is used for a justified medical/therapeutic or research purpose and (b) is in line with Islamic and medical regulation in the country (The Hashemite Kingdom of Jordan, 2014).

### Procedural Requirements and Ethical Safeguards

#### Stage of Embryonic Development “Cut-Off Date”

The cut-off dates for embryonic development after which “ethical” research can be carried out on ESCs vary considerably” (Isasi and Knoppers, 2006a, b).

The majority of Muslim scholars, however, recognize human life as beginning 40 or –120 days after conception. JSCS, in its Article (3), permits the use of fertilized eggs not older than 5 days (The Hashemite Kingdom of Jordan, 2014). The 5 days rule came as a strict approach to protect human live.

#### Source of Stem Cells

According to the Iftaa’ Law number (60) of the year 2006, and the modifying Law number (4) of the year 2009, the Board of Iftaa’, Research and Islamic Studies is the responsible entity in Jordan to issue Fatwa on emerging issues of public affairs that need Ijtihad and to review referred draft laws and regulations (Iftaa’ Department, 2019b).

Article (3) of JSCS divides stem cells based on their permissible source into the following categories (The Hashemite Kingdom of Jordan, 2014):

a. Embryonic stem cells derived from IVF starting from the date of fertilization and up to 5 days following the beginning of successive divisions.
b. Adult stem cells derived from: (i) umbilical cord blood after delivery, (ii) deciduous teeth, (iii) different human tissue after birth, or (iv) spontaneous legal abortion embryos and embryonic tissue inside the uterus or umbilical cord or placenta, and its tissue, amniotic fluid whether inside or outside the uterus.
c. Human induced pluripotent stem cells (iPSCs), which are a type of pluripotent stem cells generated directly from adult cells provided they are not implanted into a woman’s uterus and/or used for reproductive purposes.
d. Stem cells created through somatic cell nuclear transfer (SCNT) into unfertilized human eggs or zygotes provided it is not implanted into a woman’s uterus and/or used for reproductive purposes.
e. Stem cells created through the transfer of human somatic cell nuclei into non-human animal eggs provided it is not implanted into a woman’s uterus and/or used for reproductive purposes.

Additionally, Article (9) of JSCS prohibits the procurement of stem cells unless it is from one of the permissible sources according to Shari’ah and defined by Ijtihad made by the scholars of the General Iftaa’ Department of Jordan (The Hashemite Kingdom of Jordan, 2014). The above divisions are relatively similar to the ones announced by the Board of Iftaa’, Research and Islamic Studies during its ninth session in Amman, Jordan (03 October 2013) (Board of Iftaa’ R and IS, 2014). However, one essential difference is source (d): stem cells created through SCNT into unfertilized human eggs or zygotes, which is included as a permissible source in the JSCS but not included as a permissible source in the announcement of the Board of Iftaa’, Research and Islamic Studies (Board of Iftaa’ R and IS, 2014; The Hashemite Kingdom of Jordan, 2014).

#### Creation of Embryos Specifically for Research

Embryos created specifically to be used in scientific research are the most problematic source of stem cells. Some claim that the use of spare embryos is less controversial than those created specifically for research purposes (Herder, 2002). Spare embryos (i.e., supernumerary embryos) are embryos produced for reproductive purposes and no longer needed, thus will be discarded. Using such embryos for research is generally considered less controversial than creating new “potential human beings” destined to be destroyed and used for research purposes. Additionally, in IVF programs, some oocytes may not develop adequately to be implanted or fail to fertilize in the first place, such products can also be donated for use in research (Campbell, 2005). The JSCS is silent about this issue. It does not specifically mention whether or not creating embryos specifically for research purposes is allowed. However, the Fatwa of the Board of Iftaa’, Research and Islamic Studies clearly states that ESCs cannot be obtained from embryos created through IVF specifically for research purposes. Additionally, the Fatwa does not list ESCs obtained from SCNT as a permissible source as mentioned earlier (Board of Iftaa’ R and IS, 2014).

#### Protection of Donors of Blastocysts, Gametes, Embryos, and Somatic Cells

The protection of the rights of donors of blastocysts, gametes, embryos, and somatic cells is addressed in most jurisdictions. These safeguards include the requirement of free and informed consent, measures to ensure confidentiality and privacy, prohibition of financial gains or incentives for the donation, and prohibition of conflicts of interest between researchers and medical staff. Each of these will be expanded below.

(1) Free and informed consent: With the advent of the Nuremburg Code, informed consent became a primary requirement for research involving human subjects. Informed consent has gained greater importance in research involving stem cells (Lo et al., 2004). This includes the need for written informed consent, disclosure of material information (Kington, 2016) (including researchers’ financial interests), and provisions for withdrawal of consent (see Table 2).

**TABLE 2:** Example of Information to be disclosed during the Consent Process for Embryo Donor(s) (Kington, 2016).

**Information to be disclosed during the consent process:**
• Donation is voluntary and will not affect any healthcare service provided.
• The embryos will be used to derive ESCs for research.
• What will happen to the embryos during the derivation of ESCs for research.
• HESCs derived from the embryos might be kept for many years.
• Donation is made without any restriction or direction regarding the individual(s) who may receive medical benefit from the use of the ESCs, such as who may be the recipient(s) of cell transplants.
• The research is not intended to provide direct medical benefit to the donor(s).
• The results of research using the ESCs may have commercial potential; however, the donor(s) will not receive financial or any other benefits from any such commercial development.
• What potentially identifying information about the donor(s) will be available to researchers.
• Donor(s) retain the right to withdraw consent for the donation of the embryo until the embryos are actually used to derive ESCs or until information which could link the identity of the donor(s) to the embryo are no longer retained, if applicable.
• No payments, cash or in kind, will be offered for the donated embryos.

In alignment with other laws and regulations, the JSCS, in its Article (8), prohibits the procurement and use of stem cells regardless of the source, unless a written informed consent is obtained from the donor or one of the parents or the legal guardian on a form issued by the Minister of Health (The Hashemite Kingdom of Jordan, 2014).

(2) Conflict of interest between the medical staff and the researchers: To ensure that the treating physician has no undue influence on patients to donate their embryos, it is recommended that the treating physician neither be involved in the conduct of ESC research nor be the one who consents the donor (Lo et al., 2003, 2004; Caulfield et al., 2007; Lo and Parham, 2009). The JSCS completely overlooks the issue of the potential for conflict of interest between medical staff and researchers involved in ESC research (The Hashemite Kingdom of Jordan, 2014).

(3) Financial gain: Reimbursement to donors for out-of-pocket expenses is ethically acceptable as it does not provide any financial advantage to the donor for his/her participation. Nonetheless, offering larger amounts of out-of-pocket payment (e.g., remuneration) to donors is debatable (Spar, 2007).

Article (6) of the JSCS prohibits compensation or financial gain from stem cell donation. In order to avoid any exploitation, Article (6) also mentions that the Minister of Health determines the conditions where donation of stem cells is prohibited in order to protect the general public well-being (The Hashemite Kingdom of Jordan, 2014).

(4) Confidentiality and privacy: Confidentiality must be tightly regulated to alleviate potential stigmatization or discrimination. Donor/participant identifiers must be kept in case needed for audits by the regulatory bodies for the approval process of new treatments. However, identifiers will be replaced by a unique code to preserve confidentiality. Uncertainties about confidentiality sometimes discourage donors from approving to be re-contacted (Lo and Parham, 2009).

Under Article (15) of JSCS, all data and records in stem cell banks are confidential, and shall not be disclosed unless under conditions deemed by the enacted legislation (The Hashemite Kingdom of Jordan, 2014). However, it fails to explicitly ensure provisions for protecting privacy and maintaining confidentiality in the context of stem cell research. Files with donors’/participants’ identities should be protected through tight security measures (e.g., unique code). Computers used to store such files must be password-protected, not connected to the internet and stored in a secure room with access limited to selected personnel from the research team using a card-key or equivalent system to record each entry and monitor any inappropriate access.

### Requirements for Research Governance

#### National Authority/Body Responsible

There is a general consensus on the need for regulatory oversight of assisted reproductive technologies in most jurisdictions. However, there is no consensus on the type of regulatory structure (Campbell, 2005).

The development of a centralized, independent, and transparent public authority mandated to award licenses and provide oversight of stem cell research activities is fundamental to ensure responsible governance of science (Isasi and Knoppers, 2006b).

According to Article (4) of JSCS, the Minister of Health establishes the “National Committee for Stem Cells.” Of note, most of the Committee’s responsibilities and duties (Article 5) focus on drafting and modifying the Statute as well as laying out specific regulations for stem cell banking in accordance with international standards. Surprisingly, nothing is mentioned about oversight of stem cell use in research (The Hashemite Kingdom of Jordan, 2014).

#### Requirement for Monitoring Activities

To ensure good clinical and laboratory practices, monitoring activities are needed. Article (19) of the JSCS states that based on the delegation of the National Committee for Stem Cells, the Minister of Health issues the licensing requirements for stem cell banks including technical standards, equipment requirements, and qualifications of the employees and their expertise. In addition, the Minister of Health is responsible for the overall quality management design of the bank, including quality management programs, as well as confidentiality and safety measures needed to ensure the safety and integrity of the samples (The Hashemite Kingdom of Jordan, 2014). However, well-defined monitoring activities or assessment of compliance with the legislation for research involving human embryos and stem cells are not mandated under the Statute.

#### Institutional Oversight of Stem Cell Research: Ethics Review and Approval

As mentioned before, stem cell research must show scientific validity to justify the use of human embryos and stem cells. Stem cell research may raise some ethical concerns that are beyond the capacity or scope of Institutional Review Boards/Research Ethics Committees (IRBs/RECs), as members often lack the expertise and training to review the highly specialized stem cell research projects. Moreover, some ethical issues related to stem cell research are related to other ethical issues including the consequences of transplanting human stem cells into non-human animals, which may result in transferring unique human traits into animals, such as human cognition (Campbell, 2005; Scott, 2008; Caulfield et al., 2015).

A highly specialized stem cell research oversight (SCRO) committee is needed at each institution. It should be comprised of basic scientists, IVF physicians, embryologists, legal and ethics experts in addition to patient advocates and community representatives. It would be responsible for the review, approval, and oversight of stem cell research, and should work closely with the IRB/REC when human participants are involved. Examples include “Embryonic Stem Cell Research Oversight Committees” (ESCROs) in the United States, “Stem Cell Oversight Committee” (SCOC) in Canada, and “Institutional Committees for Stem Cell Research” (IC-SCR) in India (Campbell, 2005; Zettler et al., 2007; Scott, 2008; Caulfield et al., 2015).

Many jurisdictions have adapted this “dual review” approach to guarantee that such complex and highly controversial research is aligned with certain requirements related to stem cell use in research. These include: the prohibition of reproductive cloning, the creation of chimeras, and human germ-line modification, as well as the source of stem cells and the informed consent of embryo/gamete donors (Caulfield et al., 2015).

The JSCS is silent on the approvals required before stem cells can be imported and used in medical research within institutions. More specifically the need for review and approval of stem cell research protocols by an IRB/REC is not addressed.

#### Type of Institute Allowed to Conduct Stem Cell Research

Very few jurisdictions, if any, have set limitations on the type of institution allowed to conduct stem cell research. The private sector usually prioritizes research with high potential of financial gain and non-profit organizations focus their research on a specific disease or demographic according to their scope of work. These differences in funding priorities can create social justice issues. On the contrary, funding decisions in the government and public sector are usually guided by the health needs of the society minimizing such injustice (Dresser, 2006).

Article (9) of the JSCS prohibits the collection, procurement, storage, or use of ESCs for therapy or research, unless executed by a specialized governmental institution or publicly funded academic institution (The Hashemite Kingdom of Jordan, 2014), for example, public universities and governmental research centers.

#### Criminal Sanctions

Good governance calls for provisions that contemplate sanctions and enforcement mechanisms. A survey on ESC and human cloning regulations, adopted in 50 countries, found that 27 countries have set statutory provisions (Isasi and Knoppers, 2006a). Nonetheless, differences in the statutory responses exist, with some considering engagement in the prohibited activities or violation of the regulatory requirements to be a criminal offense, punishable by imprisonment; while others punish transgressions only with civil fines.

Since the JSCS is under the Public Health Law, Article (18) states that punishments/sanctions specified in the Public Health Law are enforced if there is contravention of any article of the Statute (The Hashemite Kingdom of Jordan, 2014). According to Article (66) of the Public Health Law, these punishments may include imprisonment from 2 months to 1 year and/or a monetary fine from 500 to 1000 Jordanian Dinars (The Hashemite Kingdom of Jordan, 2008).

## Discussion

The JSCS is the first step in the journey of developing a comprehensive law. Although it came with the intention to produce a comprehensive Statute for stem cell therapy and research, it focused primarily on stem cell banking as the initial committee was most interested in the practical and relevant issues that existed at the time. However, it did not explicitly state that it was the duty of the Statute to stay abreast of new advances in science and to produce verdicts on any situation resulting from such new advances that could impact or affect research. Examples of new advances include: three-parent babies, CRISPR and gene editing, uterus transplantation, etc. Therefore, plans to update the Statute to address these issues are being discussed in the meantime.

In general, the JSCS permits the use of stem cells in research including ESCs under certain conditions. The JSCS appears to have adopted a policy approach that falls between restrictive and intermediate. This is expected as Jordan is one of the developing countries with advanced health care services in the region and a recent increase in the number of clinical trials. At the same time, religion and cultural factors play an important role in shaping laws and policies within the Jordanian jurisdiction (International Intellectual Property Institute [IIPI], 2004).

The JSCS integrates many of the common elements of the stem cell regulations stated in the literature. Essential aspects covered by the Statute include the justification of medical/therapeutic need or research purpose, the need for written informed consent from the donor or one of the parents or the legal guardian, and the licensing requirements for stem cell banks, including technical standards, required equipment, and qualifications/expertise of the employees.

Many important procedural requirements and ethical safeguards are also integrated in the Statute. The stage of embryonic development, with “cut-off date” set at 5 days from conception, is in line with Islamic faith. In addition, the Statute lists the permissible sources of stem cells (Article 3) aligned with fatwa by Islamic scholars (Al-Aqeel, 2009; Board of Iftaa’ R and IS, 2014).

Moreover, the JSCS prohibits compensation or financial gain from stem cell donation. Being vulnerable, financial gain may encourage women or their partners to take avoidable risks, especially those who are poorly educated and have no employment alternatives. This is consistent with the Jordanian Clinical Studies Law, which permits research participants to be compensated for their transportation fees only. The JSCS also specifies the fines and punishments applied if there is contravention of the Statute, in accordance with Jordanian Public Health Law.

Nonetheless, the JSCS is deficient in several aspects and less consistent in others.

First, when listing the permissible sources of stem cells, the Statute is very general and does not clearly include the *fatwa* for each source and the conditions under which each source is considered legitimate. Accordingly, several points may be misunderstood. For example, for source (a) (i.e., ESCs derived from IVF starting from the date of fertilization up to 5 days following the beginning of successive divisions) to be permissible, IVF must meet the following conditions: (i) fertilization must be between individuals in a valid marriage, (ii) sperm must be collected from the husband and the eggs from the wife, (iii) surrogacy is not allowed, (iv) only the number of fertilized eggs needed for reproduction purposes should be transferred to the uterus, and (v) the procedure must be performed by a competent and reliable medical team (Fadel, 2002). Furthermore, the use of stem cells derived from source (b) (i.e., adult stem cells derived from spontaneous legal abortion embryos) is allowed only under regulations that restrict deliberate abortion for the purpose of donation to research or use it as a tool to permit illegitimate abortion as it can lead to endorsing such abortions (Al-Aqeel, 2009). The only source not included in the announcement by the Board of Iftaa’, Research and Islamic Studies is source (d) (i.e., stem cells derived through SCNT). Generally, there is disagreement between Muslim Scholars on the permissibility of this source. Using SCNT includes the creation of new embryos specifically for research purposes followed by the destruction of these embryos to harvest stem cells, in addition to the use of human cloning techniques. Moreover, these same techniques could possibly be used to create human embryos, which can be implanted into a woman’s uterus and used for reproductive purposes in the absence of strict oversight and supervision (Campbell, 2005). This, however, is prohibited worldwide.

Moreover, the Fatwa by the General Iftaa’ Department of Jordan emphasizes that in order to obtain stem cells from the above-mentioned sources, it must be ensured that no harm is caused to the donors’ health. We believe that the above-mentioned points should be clearly spelled out in the Statute in order to avoid any possible misinterpretation.

Although the JSCS requires written informed consent, it fails to address important elements of the process of obtaining informed consent, especially in the context of stem cell research.

The Statute also fails to address the information that should be disclosed to participants/donors, including researchers’ financial interests, as summarized in Table 2 (Kington, 2016). Additionally, one critical ethical issue related to informed consent is whether donors comprehend key information about stem cell donation (Woodsong and Karim, 2005). Assessing comprehension is achievable and has been applied in other types of research (Lomax et al., 2007). For example, ask participants to complete a quiz-like interview after disclosure of information during the consent process (Woodsong and Karim, 2005).

Other international ethical challenges that have not been addressed by the JSCS include provisions for withdrawal of consent, requirements of informed consent for the secondary use of reproductive materials and ESCs, or possible secondary use in future research. This is very critical, especially since some donors may object to all types of stem cell research, while others are more accepting of certain types of ESC research. For example, some donors might agree to infertility research, but disagree with research involving the extraction of stem cell lines or research that may yield commercial products or patents. Literature shows that people commonly hold unique emotional and moral values when it comes to their reproductive materials, in comparison with other tissues (Lo and Parham, 2009). Furthermore, the JSCS only includes provisions for ensuring privacy of the donor and maintaining confidentiality in stem cell banking, while overlooking the issue of privacy and confidentiality of participants in the context of stem cell research.

The Statute also fails to clearly delineate the requirements for the governance of stem cell research including: (1) the national entity responsible for regulating stem cell research, as the responsibilities of the Stem Cells Committee formed by this Statute focuses on regulating stem cell banking; (2) the requirement for monitoring stem cell research activities; and (3) the institutional/ethics committee responsible for reviewing, providing recommendations, and issuing approval for the proposed stem cell research at the institution.

Defining the three above governance requirements is crucial, as allowing stem cell research to proceed without strong governance and monitoring activities could lead to devastating results and harm to individuals and the public. A recent article published in one of the Jordanian newspapers indicated that in the middle of organizational chaos and the weakness of the official control mechanisms, stem cell therapy trials are being conducted on Jordanian and Arab patients in private hospitals, clinics, and research centers in Jordan. Patients are being informed that they are receiving licensed treatment for various intractable diseases, when in fact they are receiving unlicensed treatment as part of research studies without scientific merit, oversight, or participant protections (AlKiswani, 2017).

It is clear from the Statute that only government organizations or publicly funded academic institutions are allowed to obtain, use, or store ESCs. This was driven by concerns of potential termination of pregnancies, an illegal act in Jordan unless the mother’s life or health is at risk. It can also be argued that government organizations might have higher levels of transparency as they are under supervision by the Ministry of Health and the specialized committee “National Committee for Stem Cells” (Dajani, 2014). However, it remains unclear whether the private sector is allowed to use other sources of stem cells such as somatic stem cells and stem cells produced by SCNT and iPSCs techniques (Campbell, 2005).

Other deficiencies in the JSCS include the lack of provisions to prohibit conflicts of interest between medical staff and researchers involved in ESC research, commercialization, storage, and the importation of embryos and stem cells from outside the country.

Taking into account Jordan’s status in the region as an indispensable health care provider for patients from abroad, stem cell research is a subject of current interest and high anticipation. Furthermore, Jordan is one of the few Arab countries that have national regulations to protect human subjects in clinical trials (U.S. Office for Human Research Protections, 2019). Thus, the JSCS should serve as an example for other countries in the region, emphasizing the importance of having national regulation to govern and oversee such sensitive issues (Dajani, 2014). The International Society for Stem Cell Research (ISSCR) sets international guidelines governing stem cell research. These guidelines inspire and guide organizations and researchers conducting stem cell research (International Society for Stem Cell Research, 2006, 2016). We recommend that the ISSCR should draw from and collaborate with jurists and scientists from countries where there is a majority of Muslims and who have a law or statute for stem cell research and therapy when updating the ISSC guidelines. Currently, there are no Muslims in the ethics committee of the ISSCR.

In a previous comparative study on data sharing requirements in stem cell research between the JSCS and the Framework of the Global Alliance for Genomics and Health (GA4GH) published in 2014, the JSCS was also found deficient in several other aspects. These include the lack of active participation of the public, transparency in the data sharing and access processes, ensuring the quality and safety of the data, preforming in-depth risk-benefit analysis, and maintaining sustainability of stem cell banks and registries (Al-Tabba et al., 2018). Fulfilling these requirements will foster future international collaborative work.

## Conclusion

Being the first in the Arab World to pass such legislation, the JSCS can help pave the way for public discussions and adoption of national laws aimed at protecting the welfare of the public and advancing science in other Arab countries. The Statute covers many important aspects to regulate stem cell use; yet, it pays more attention to stem cell banking in many of its articles, while overlooking many critical aspects in regulating stem cell research activities.

In light of the deficiencies presented in this article, future review and amendment of the Statute is needed. Moreover, we share lessons learned from developing the Statute that may be useful to others who wish to undertake similar efforts. Countries interested in developing future regulations and guidance surrounding stem cell research should also access and study existing guidelines and laws in other countries and organizations as a guide for developing their own law.

## Author Contributions

AA-T contributed to collection of data, analysis of data, literature review, drafting the first draft, and approval of the final draft. RD contributed to analysis of the data, literature review, critically reviewing the draft, and approval of the final draft. MA-H contributed to conception of the idea, collection of the data, critically reviewing the draft, and approval of the final draft. All authors took public responsibility.

## Conflict of Interest

The authors declare that the research was conducted in the absence of any commercial or financial relationships that could be construed as a potential conflict of interest.

## Figures and Tables

**Table:** Glossary of Islamic Terms (Al-Bar and Chamsi-Pasha, 2015b; Iftaa’ Department, 2019a,b).

**Term**	**Definition**
Board of Iftaa’, Research and Islamic Studies	The responsible entity in Jordan to issue fatwa on emerging issues of public affairs that need Ijtihad and to review referred draft laws and regulations.
Fatwa	An initially non-binding, context specific Islamic ethico-legal assessment or ruling issued by a trained Islamic jurist. The Fatwa of the highest authority in the country is binding to that country.
Faqih	A faqih is a specialist on the detailed rules of Islamic law, or fiqh.
Fiqh	Jurisprudential understanding or an ethico-legal ruling. Fiqh details the Islamic legal rulings on human actions on the basis of the Islamic legal sources. Most fiqh rulings, particularly with regard to contemporary issues, are subject to difference of opinion.
General Iftaa’ Department of Jordan	The council of Muftis who answer the questions of members of the public, whether they relate to worship or financial transactions or personal statutes.
Iftaa’	The giving of legal opinions (fatwas) by muftis.
Ijmaa	Consensus agreement, usually of the scholars; an authoritative source for Islamic law; after Qur’an and Sunna.
Ijtihad	Juristic effort or methodology used to construct a fatwa in a novel or unprecedented case. Ijtihad describes the process of deriving legal rules from their sources by qualified experts of Islamic law. It is a vital process with regard to newly emerging issues that have not been explicitly mentioned in either Qur’an or Sunnah.
International Islamic Fiqh Academy of Organization of Islamic Conferences (OIC-IFA)	Majma Al-Fiqhi Al-Islami of the Organization of the Islamic Conference, Jeddah.
Islamic Fiqh Council	An affiliate of the Muslim World League with an independent legal personality. It was founded by a resolution of the Constituent Council in the year 1398 AD, and is made up of a select group of Muslim jurists and scholars who meet periodically to consider serious issues concerning the Muslim Ummah and issue appropriate rulings based on the texts of the Holy Qur’an and the immaculate Sunnah.
Mufti	A Mufti is a qualified person who gives legal verdicts (answers) to particular questions. These questions could be new, as is the case in most biomedical
	advancements. In this case, the Mufti needs to be qualified to carry out ijtihad.
Muslim World League	An international non-governmental Islamic organization based in the Holy City of Makkah.
Organization of the Islamic Conference (OIC)	An intergovernmental organization representing 57 Muslim states, Jeddah.
Qur’an	God’s revelation to the Prophet Muhammad; the most authoritative document in Islam.
Shari’ah	Islamic law; the correct path of action as determined by God.
Sunnah or (Sunna)	The established practice of the Prophet Muhammad (peace be upon him), transmitted through reports (hadith) on his sayings, actions, or tacit approval. The Sunnah is the second authoritative source of law after the Qur’an.
	

## References

[B1] Abumaree M. H. Askar A. S. Al Kalionis B. Abomaray F. M. Jawdat D. Hajeer A. H. (2014). Stem cell research and regenerative medicine at king abdullah international medical research center. *Stem Cells Dev.* 23 12–16. 10.1089/scd.2014.0303 25457954

[B2] Al Arabiya English (2020). *Coronavirus: UAE Develops COVID-19 Treatment, ‘Could be Game-Changer’ | Al Arabiya English [Internet].* Available online at: https://english.alarabiya.net/en/coronavirus/2020/05/01/Coronavirus-UAE-develops-COVID-19-treatment-could-be-game-changer- (accessed May 19, 2020).

[B3] Al-Aqeel A. I. (2005). Ethical guidelines in genetics and genomics. An Islamic perspective. *Saudi Med. J.* 26 1862–1870. 16380763

[B4] Al-Aqeel A. I. (2009). Human cloning, stem cell research: an Islamic perspective. *Saudi Med J.* 30 1507–1514. 19936411

[B5] Al-Bar M. A. Chamsi-Pasha H. (eds) (2015a). “Chapter 3: the origins of islamic morality and ethics,” in *Contemporary Bioethics: Islamic Perspective*, (New York: Springer International), 58–66. 29809384

[B6] Al-Bar M. A. Chamsi-Pasha H. (2015b). *Glossary in Contemporary bioethics: Islamic Perspective.* New York: Springer International, 261–267. 29809384

[B7] AlKiswaniH. (2017). *Stem Cell Ciaos in the Medical Field. Alghad Newspaper [Internet].* Available online at: https://alghad.com/ 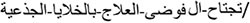 /(cited October 7, 2019).

[B8] Al-Tabba A. Al-Omari A. Al-Hussaini M. (2018). “Appraisal of the Jordanian law for data sharing in stem cell research: in the light of the” GA4GH Framework” for innovative cancer care,”,” in *Proceedings of the 2018 1st International Conference on Cancer Care Informatics (CCI)*, Amman, 232–235.

[B9] Arab Scientific Community Organization (2014). 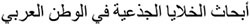 Available online at: http://arsco.org/article-detail-456-3-0 (accessed May 18, 2020).

[B10] Board of Iftaa’ R and IS (2014). *Ruling No. 189 (9/2013) Stem Cells types and Islamic Ruling [Internet].* Available online at: http://aliftaa.jo/Decision.aspx?DecisionId=237 (accessed October 7, 2019).

[B11] Campbell A. (2005). Ethos and economics: examining the rationale underlying stem cell and cloning research policies in the United States, Germany, and Japan. *Am. J. Law Med.* 31 47–86. 10.1177/009885880503100102 15895815

[B12] Caulfield T. Kamenova K. Ogbogu U. Zarzeczny A. Baltz J. Benjaminy S. (2015). Research ethics and stem cells: is it time to re-think current approaches to oversight? *EMBO Rep.* 16 2–6. 10.15252/embr.201439819 25476708 PMC4304722

[B13] Caulfield T. Ogbogu U. Isasi R. M. (2007). Informed consent in embryonic stem cell research: are we following basic principles? *Can. Med. Assoc.* 176 1722–1725. 10.1503/cmaj.061675 17548387 PMC1877848

[B14] Dajani R. (2011). *Islam and Stem Cell Ethics Talk at the Belief in Dialogue Conference.* Sharjah: American University of Sharjah.

[B15] Dajani R. (2014). Jordan’s stem-cell law can guide the Middle East. *Nature* 510:189. 10.1038/510189a 24919888

[B16] Dajani R. (2017). “Ethics of stem cell research in the Arab Region,” in *Research Ethics in the Arab Region*, ed. Silverman H. (Cham: Springer), 107–116. 10.1007/978-3-319-65266-5_10

[B17] Dresser R. (2006). Private-sector research ethics: marketing or good conflicts management? The 2005 john j. conley lecture on medical ethics. *Theor. Med. Bioeth.* 27 115–139. 10.1007/s11017-005-5289-5 16699963

[B18] Editor H. S. Edwards S. (2017). *Research Ethics in the Arab Region.* Cham: Springer, 1–390.

[B19] El-Awady N. (2008). *Gulf States Embrace Stem Cell Technologies At Home and Abroad [Internet]. Nature Reports Stem Cells.* Berlin: Nature Publishing Group.

[B20] Fadel H. E. (2002). The Islamic viewpoint on new assisted reproductive technologies. *Fordham Urban Law J.* 30:147. 15868666

[B21] Fadel H. E. (2012). Developments in stem cell research and therapeutic cloning: islamic ethical positions, a review. *Bioethics* 26 128–135. 10.1111/j.1467-8519.2010.01840.x 21039687

[B22] Flynn J. M. Matthews K. R. W. (2010). Stem cell research in the Greater Middle East: the importance of establishing policy and ethics interoperability to foster international collaborations. *Stem Cell Rev. Rep.* 6 143–150. 10.1007/s12015-010-9133-6 20198516

[B23] Gottweis H. Salter B. Waldby C. (2009). “Globalization, stem cell markets and National interests,” in *The Global Politics of Human Embryonic Stem Cell Science*, eds Webster A. Wyatt S. (New York: Springer), 22–34.

[B24] Herder M. (2002). The UK model: setting the standard for embryonic stem cell research? *Health Law Rev.* 10:14. 15739306

[B25] Iftaa’ Department (2019a). *About the General Fatwa Department [Internet].* Available online at: https://www.aliftaa.jo/ShowContentEn.aspx?Id=74 (accessed October 6, 2019).

[B26] Iftaa’ Department (2019b). *Board of Ifta’, Islamic Studies and Research [Internet].* Available online at: https://aliftaa.jo/ShowContentEn.aspx?Id=78 (accessed October 7, 2019).

[B27] International Intellectual Property Institute [IIPI] (2004). *Jordan Pharmaceutical Industry Report Released [Internet].* Washington, DC: IIPI.

[B28] International Society for Stem Cell Research (2006). *Guidelines for the Conduct of Human Embryonic Stem Cell Research.* Skokie, IL: International Society for Stem Cell Research.

[B29] International Society for Stem Cell Research (2016). *Guidelines for Stem Cell Research and Clinical Translation.* Skokie, IL: International Society for Stem Cell Research.

[B30] Isasi R. Knoppers B. (2006a). R Mind the gap: policy approaches to embryonic stem cell and cloning research in 50 countries. *Eur. J. Health Law* 13 9–25. 10.1163/157180906777036328 16838747

[B31] Isasi R. M. Knoppers B. M. (2006b). RM Beyond the permissibility of embryonic, and stem cell research: substantive requirements, and procedural safeguards. *Hum. Reprod.* 21 2474–2481. 10.1093/humrep/del235 16877375

[B32] Kington R. S. (2016). *National Institutes of Health Guidelines for Human Stem Cell Research [Internet].* Bethesda, MD: NIH.

[B33] Levine A. D. (2008). Identifying under-and overperforming countries in research related to human embryonic stem cells. *Cell Stem Cell.* 2 521–524. 10.1016/j.stem.2008.05.008 18522844

[B34] Lo B. Chou V. Cedars M. I. Gates E. Taylor R. N. Wagner R. M. (2003). Medicine. Consent from donors for embryo and stem cell research. *Science* 30:921. 10.1126/science.1087038 12920284

[B35] Lo B. Chou V. Cedars M. I. Gates E. Taylor R. N. Wagner R. M. (2004). Informed consent in human oocyte, embryo, and embryonic stem cell research. *Fertil. Steril.* 82 559–563. 10.1016/j.fertnstert.2004.01.040 15374695

[B36] Lo B. Parham L. (2009). Ethical issues in stem cell research. *Endocr. Rev.* 30 204–213. 19366754 10.1210/er.2008-0031 PMC2726839

[B37] Lomax G. P. Hall Z. W. Lo B. (2007). Responsible oversight of human stem cell research: the California Institute for Regenerative Medicine’s medical and ethical standards. *PLoS Med.* 4:e114. 10.1371/journal.pmed.0040114 17488179 PMC1858709

[B38] Matsumoto M. M. Dajani R. Matthews K. R. W. (2015). Cord blood banking in the Arab world: current status and future developments. *Biol. Blood Marrow Transplant.* 21 1188–1194. 10.1016/j.bbmt.2015.01.012 25687797

[B39] Muslim World League (2020). 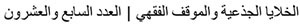 Available online at: https://www.eajaz.org/index.php/component/content/article/85-Number-xxvii/791-Stem-cells-and-the-doctrinal-position (accessed May 19, 2020).

[B40] National Committee of BioEthics [NCBE] (2016). *Implementing Regulations of the Law of Ethics of Research on Living Creatures*, Second Edn Available online at: https://prod.kau.edu.sa/Med/ali/files/Publications/Guide/National_Committe_of_BioEthics-Regulations_of_the_Law_of_Ethics_of_Research_on_Living_Creatures.pdf (accessed 19 May 2020).

[B41] National Human Genome Research Institute (2017). *Cloning Fact Sheet | NHGRI [Internet].* Available online at: https://www.genome.gov/about-genomics/fact-sheets/Cloning-Fact-Sheet (accessed October 6, 2019).

[B42] Scott C. T. (2008). Stem cells: new frontiers of ethics, law, and policy. *Neurosurg. Focus.* 24:E24. 10.3171/FOC/2008/24/3-4/E23 18341401

[B43] Spar D. (2007). The egg trade—making sense of the market for human oocytes. *N. Engl. J. Med.* 356 1289–1291. 10.1056/nejmp078012 17392298

[B44] The Hashemite Kingdom of Jordan (2008). *Law No.47 of 2008 on Public Health.* Jordan: The Hashemite Kingdom of Jordan.

[B45] The Hashemite Kingdom of Jordan (2014). *Statute No. 10 of 2014 on Stem Cell.* Jordan: The Hashemite Kingdom of Jordan, 172–179.

[B46] U.S. Office for Human Research Protections (2019). *Department of Health and Human Services PURPOSE. International Compilation of Human Research Protections [Internet].* Available online at: http://www.hhs.gov/ohrp/international/ (accessed 07 October 2019).

[B47] UNESCO Cairo Office (2011). *Ethics and Law in Biomedicine and Genetics; an Overview of National Regulations in the Arab States.* Paris: UNESCO.

[B48] UNGAOR (2005). *Resolutions Adopted by the General Assembly at its 59th Session [Internet].* Available online at: http://research.un.org/en/docs/ga/quick/regular/59 (accessed October 7, 2019).

[B49] Wadvalla B.-A. (2012). *Stem Cell Research and Islamic Regulations [Internet].* Available online at: https://www.natureasia.com/en/nmiddleeast/article/10.1038/nmiddleeast.2012.125 (accessed May 18, 2020).

[B50] Walters L. (2004). Human embryonic stem cell research: an intercultural perspective. *Kennedy Inst. Ethics J.* 14 3–38. 10.1353/ken.2004.0019 15250112

[B51] Woodsong C. Karim Q. A. (2005). A model designed to enhance informed consent: experiences from the HIV prevention trials network. *Am. Public Health Assoc.* 95 412–419. 10.2105/ajph.2004.041624 15727968 PMC1449193

[B52] Yahia M. (2013). *Specials: Making The Most of a Head Start in Stem Cell Research [Internet].* Available online at: https://www.natureasia.com/en/nmiddleeast/article/10.1038/nmiddleeast.2013.212 (accessed May 18, 2020).

[B53] Zettler P. Wolf L. E. Lo B. (2007). Establishing procedures for institutional oversight of stem cell research. *Acad. Med.* 82 6–10. 10.1097/01.acm.0000250025.17863.bf 17198282

